# Itaconate: A Metabolite Regulates Inflammation Response and Oxidative Stress

**DOI:** 10.1155/2020/5404780

**Published:** 2020-07-17

**Authors:** Ruidong Li, Peng Zhang, Yaxin Wang, Kaixong Tao

**Affiliations:** ^1^Department of Gastrointestinal Surgery, Union Hospital, Tongji Medical College, Huazhong University of Science and Technology, Wuhan 430022, China; ^2^Department of Critical Care Medicine, Union Hospital, Tongji Medical College, Huazhong University of Science and Technology, Wuhan 430022, China

## Abstract

Metabolic products can lead to crucial biological function alterations. Itaconate is probably the best example of how a metabolic process can be diverted to generate an immunomodulator effect in macrophages. Through inflammatory stimuli, such as lipopolysaccharide, the immune response gene 1 is activated and promotes the production of itaconate from the tricarboxylic acid cycle by decarboxylating cis-aconitate. Itaconate has been reported to have multiple immunoregulatory and antioxidative effects. In addition, reports have described its antibacterial and protumor effects. The involved mechanism in these effects includes the activation of nuclear factor E2-related factor 2 by alkylation of Kelch-like ECH-associated protein 1, inhibition of aerobic glycolysis by targeting glyceraldehyde-3-phosphate dehydrogenase and fructose-bisphosphate aldolase A, inhibition of succinate dehydrogenase, and blockade of I*κ*B*ζ* translation. All of these discoveries elucidated the transformation of the pro- into anti-inflammatory status in macrophages, which is crucial in innate immunity and set the ground for the emerging therapeutic implications of itaconate. In this review, we point out that itaconate is a novel and pivotal metabolic determinant of the immunoregulatory response in macrophages and highlight studies that have improved our understanding of the connection between the immune response and metabolism. In addition, we shed light on the therapeutic potential of itaconate and its derivatives to treat inflammatory diseases.

## 1. Introduction

A growing number of reports have shown that the metabolism is widely associated with immune response and many pathologies, such as inflammatory diseases and cancer. The shift of the immune cells' function is always accompanied by markedly metabolic changes to face the energetic needs of the cells according to the circumstances [1]. Macrophages, one of the most critical innate immune cells, can assume the classical proinflammatory (M1) and proresolving (M2) macrophage phenotypes. Typically, the activation of Toll-like receptors (TLR) by bacterial lipopolysaccharide (LPS) or cytokines, such as TNF and IFN-*γ*, leads to the generation of the M1 phenotype. M1 macrophages are highly associated with tissue damage and impairment of wound healing. In contrast, the M2 phenotype, which exerts tissue repair properties, can be induced by stimulation of glucocorticoids, immune complexes, and cytokines, such as IL-4, IL-13, and IL-10, leading to the production of TGF-*β*, insulin-like growth factor (IGF), and a high increase in the expression of CD206. In the tissue microenvironment, macrophages can rapidly perceive pathogens and assume a highly inflammatory phenotype, and then shift to a less immunoactive form to decrease injury and enhance self-repair [2]. The mechanism underlying the phenotype transition of M1 to M2 still needs further exploration. However, metabolic reprogramming is undoubtedly involved in this phenotype transition [2, 3]. The M1-type macrophages display increasing glycolysis and decreasing oxidative phosphorylation, which is considered to promote the production of reactive oxygen species (ROS), tumor necrosis factors-*α* (TNF-*α*), interleukin-1*β* (IL-1*β*), and other proinflammatory mediators. Glycolysis can provide energy rapidly, which meets the needs of M1 macrophages to react immediately during an infection. In contrast, M2-type macrophages show enhanced oxidative phosphorylation and oxidative metabolism. The M2 macrophage is associated with long-term tissue repairing processes, and thus, oxidative metabolism, which comprises several steps to generate energy efficiently, is the best option [4].

**Figure 1:**
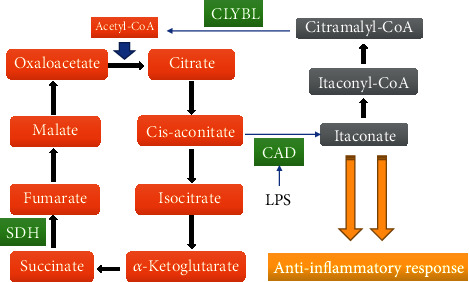
Metabolism of itaconate. Itaconate is produced from TCA. An inflammatory stimulus such as LPS promotes the expression of *Irg1*, which transforms cis-aconitate to itaconate in the mitochondrial matrix. Itaconate is converted to itaconyl-CoA, and then citramalyl-CoA. Citramalyl-CoA is catalyzed to pyruvate and acetyl-CoA by citrate lyse subunit beta-like (CLYBL). Acetyl-CoA will be reused by the TCA.

The tricarboxylic acid cycle (TCA) is the most common metabolic pathway in aerobic organisms (Figure 1). The TCA cycle provides not only the most efficient production of adenosine triphosphate (ATP) but also several intermediate metabolites, such as citrate, fumarate, *α*-ketoglutarate, and succinate, which can alter cellular functions by affecting many signaling pathways. Itaconate, a diverted derivative from the TCA cycle, was recently found to have a striking immune-regulated function. In macrophages, itaconate can be induced by a wide range of factors, such as LPS, type I and type II interferons, and agonists of Toll-like receptor (TLR) [5, 6]. All of these factors mentioned above can increase the level of enzyme aconitate decarboxylase 1 (*Acod1*), which is encoded by the immune responsive gene 1 (*Acod1*, also known as *Irg1*), thus enhancing the probability that cis-aconitate will be diverted away from the TCA cycle and promoting the production of itaconate [5]. Accumulated reports have evidenced that itaconate is a critical immunosuppressive metabolite. Additionally, many recent studies have described itaconate as an antibacterial metabolite *via* the inhibition of the activity of isocitrate lyase, which is a crucial enzyme to support the growth of bacterial during infection [6–8]. Moreover, it was shown that itaconate mitigates reperfusion injury *via* the inhibition of succinate dehydrogenase (SDH) and the induction of the antioxidative stress response [9]. All of these results significantly broadened our knowledge of itaconate. They illustrated how a metabolic process can be diverted to produce a metabolite that has a critical effect on biological functions. A full understanding of the metabolic process and the biological functions of itaconate could pave the way to therapeutic approaches in the treatment of inflammatory diseases. In this review, we describe the metabolic change of itaconate in macrophages and summarize its potential and therapeutic roles in the treatment of inflammatory diseases.

## 2. Itaconate History and Metabolism

Itaconate was first synthesized in 1836 by a chemist named Samuel Baup *via* the thermal decomposition of citric acid [10]. It was noted by Krebs in his TCA cycle investigation and, unlike succinate and malate, was found to have no significant function in supporting the respiration reaction. Therefore, for an extended period, itaconate was mainly used in industrial polymer synthesis and was always considered as of little significance in mammalian metabolism. Several years ago, itaconate was also identified as an inhibitor of bacterial growth. In addition, its antibacterial property was studied and analyzed by many scientists. Itaconate inhibits the growth of *Pseudomonas indigofera* in the absence of glucose [7]. Mechanically, this inhibiting effect was attributed to the ability of itaconate as an inhibitor of the bacterial enzyme isocitrate lyase [8, 11]. In recent years, many reports have supported the notion that itaconate can decrease the growth of a variety of bacteria by deprivation of glucose [5, 12]. Moreover, a study showed that macrophages restricted intracellular bacterial pathogens, such as *Legionella pneumophila*, *via* modifying the proteome of bacterial vacuoles and established the bactericidal role of itaconate acid in the bacterial vacuole [12]. The mechanism is that itaconate can inhibit isocitrate lyase, which is an essential enzyme for the metabolism of bacteria [8, 13]. Of note, the bacteria that lack isocitrate lyase were also inhibited by itaconate by inactivation of propionyl-CoA carboxylase [14]. However, itaconate cannot interfere with the growth of bacteria under a high concentration of glucose. Thus, it seems that itaconate exerts its antibacterial effect when acetate serves as the main energy source. In addition to the antibacterial role of itaconate, a recent study showed an emerging role of itaconate as an antivirus in neurons. Overexpression of *Acod1* restricted the replication of West Mile virus in primary cortical neurons. In a murine model of the ZIKA virus (ZIKV) infection, a novel receptor-interacting protein kinase (RIPK) signaling was found to be associated with the anti-ZIKA response in neurons. Deletion of *Ripk3* in mice increased the virial load and reduced *Acod1* expression, which implied that the antiviral effect is associated with *Acod1* [15]. Accordingly, the deletion of *Acod1* showed higher mortality and viral load in the ZIKA infection model. Moreover, 4-OI and dimethyl malonate (an SDH inhibitor) can reduce the ZIKA viral load in the brain. Thus, itaconate generated a metabolic state that inhibited the replication of the virus in the neural system by suppression of SDH during infection [15]. All of these studies illustrate various properties of itaconate as an antibacterial and antiviral agent.

Interestingly, it was found that fungi can produce itaconate, and macrophages could produce itaconate under LPS and bacterial stimulation [5, 16]. Conversely, many bacteria, such as *Yersinia pestis* and *Pseudomonas aeruginosa*, can decompose large amounts of itaconate and convert itaconate into pyruvate and acetyl-CoA, which promotes bacterial survival in macrophages [17]. A recent study showed that *Mycobacterium tuberculos*i*s* (*Mtb*) could dissimilate itaconate by Rv2498c, which is a bifunctional enzyme also involved in the L-leucine catabolism. The deletion of Rv2498c in *Mtb* resulted in the mitigation of the severity of infection in a murine model [18]. However, another study also showed that itaconate was increasingly detected in the lung tissue of *Mtb*-infected mice [19]. Therefore, these studies reveal the complex long-term evolution logic of competition and adaption among different interacting organisms. Also, further functions of itaconate in bacteria need to be explored. Recently, the elevation of itaconate synthesis was found to occur during macrophage activation. One study showed that itaconate upregulation was detected in RAW264.7 when using capillary electrophoresis time-of-flight mass spectrometry to analyze changes of metabolites during inflammatory activation [20]. Similarly, another report showed that itaconate is a metabolite generated from the TCA cycle metabolism, which was previously unidentified during macrophage activation [21]. All of these reports reveal that LPS-induced itaconate in macrophages exerts critical anti-inflammatory features, which is a novel immunoregulatory function.

The metabolism of itaconate is connected with the TCA cycle, which is a complex and basic biological process (Figure 1). A wide range of enzymes and chemicals are involved in the TCA cycle to generate reducing equivalents used by the oxidative phosphorylation to produce ATP. To synthesize itaconate, first citrate is transformed to cis-aconitate by aconitate hydratase 2. Then, cis-aconitate is catalyzed by cis-aconitate decarboxylase (CAD), encoded by *Acod1* (identified in 1955), which is responsible for the generation of itaconate in macrophages [5]. To understand how itaconate is synthesized, the crystal structures of CAD were recently explored. This study identified eight active sites critical for CAD function and crucial amino acids that make up the active center [22]. Although rare human mutations were found in the active center that could eradicate CAD activity, clarification of the CAD structure would shed light on further analysis of CAD mutations and their links to disease risks and therapeutic intervention [22]. LPS can effectively enhance the synthesis of itaconate by activating *Acod1*. Silencing *Acod1* significantly reduces itaconate levels in LPS-stimulated RAW264.7 cells (a mouse macrophage cell line). In contrast, the synthesis of itaconate is markedly enhanced by overexpression of *Acod1* in the lung cancer cell line A549 [5]. Apart from that, pyruvate dehydrogenase is partly associated with the biosynthesis of itaconate. Pyruvate dehydrogenase kinase 1 increases the phosphorylation of pyruvate dehydrogenase and consequently suppresses its activity [23]. LPS can inhibit the activity of pyruvate dehydrogenase kinase 1, which leads to more conversion of pyruvate to acetyl-CoA *via* the activation of pyruvate dehydrogenase [24]. Acetyl-CoA is a critical precursor for the production of citrate, and sufficient citrate is essential for the synthesis of itaconate. To eradicate itaconate in LPS-induced RAW264.7 cells, itaconate is first converted to itaconyl-CoA. Then, itaconyl-CoA is catabolized *via* citramalyl-CoA lyase, which is highly conserved and localized in the mitochondria, to produce pyruvate and acetyl-CoA [25–27]. The latter can be reused in the TCA cycle. All of these reactions illustrate the metabolic cycle in macrophages. Whether other metabolic routes of itaconate exist still needs further exploration.

## 3. Anti-Inflammatory Roles of Itaconate and Its Derivatives

Although itaconate was discovered in 1836, its properties in the regulation of inflammation were revealed only in 2016. Deleting *Acod1* (*Irg1*) in mice led to the elevation of proinflammatory cytokine production during macrophage activation [28]. Moreover, the lack of *Acod1* increased mortality and lung inflammation measured by the neutrophil influx and the level of cytokines in a murine model of *Mtb* infection [29]. This indicated that itaconate is critical in the control of *Mtb*-induced immunopathology. Itaconate is transported from the mitochondria to the cytoplasm, where it shows its functions *via* the carriers that transport dicarboxylate and citrate [30]. It has been reported that the activation of M1 macrophages is partially inhibited *in vitro*, and proinflammatory mediators, such as IL-6, IL-12p70, and IL-1*β*, decrease under the administration of dimethyl itaconate (DI) in LPS-treated murine bone marrow-derived macrophages (BMDMs) [28]. This study demonstrated that the anti-inflammatory effect of itaconate was irrelevant to the NF-*κ*B pathway as the LPS-induced production of TNF-*α* was not affected in the *Acod1* knockout or DI-treated macrophages [28]. Administration of DI in a mouse model of psoriasis showed the mitigation of IL-17-I*κ*B*ζ*-driven skin damage [31]. Besides DI, 4-octyl itaconate (4-OI), which can be hydrolyzed to itaconate, has been reported to have anti-inflammatory properties. 4-OI reduced IL-1*β* activity in LPS-treated mouse macrophages and showed an anti-inflammatory effect under LPS administration *in vivo* [30]. Moreover, 4-OI decreased proinflammatory cytokine production in human macrophages and systemic lupus erythematosus patient-derived PBMCs *via* activation of the *Nrf2* pathway [32]. In addition, 4-OI reduced the cytosolic nucleic acid sensing-induced inflammatory response by inhibiting STING-dependent type I IFN expression *via* activating *Nrf2* [33]. All of these studies supplied direct evidence that itaconate is a novel and promising anti-inflammatory metabolite in limiting immunopathology.

**Figure 2:**
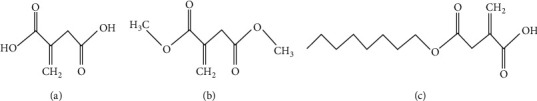
Itaconate and its derivatives. The structural formula for itaconate (a), DI (b), and 4-octyl itaconate (c).

So far, a transporter or receptor that can transport exogenous itaconate into the cytoplasm has not been identified. It was observed that extracellular itaconate could enter into the cytoplasm of adipocytes and macrophages [26, 34]. However, normally, cells need to be exposed to a high concentration of itaconate for 48-72 h, while a shorter exposure showed no such effect [34]. Thus, scientists structurally designed membrane-permeable mimics such as DI and 4-OI, both of which can permeate into cells without transporters (Figure 2). However, itaconate derivatives may not fully represent the endogenous itaconate. The alteration of structure will lead to extra effects that may not be attributed to itaconate. To understand these effects, the electrophilicity of itaconate and its derivatives should be mentioned. Itaconate, as an *α*,*β*-unsaturated carboxylic acid, is electrophilic, which allows it to accept an electron pair from nucleophiles. Thus, it can behave as an electrophilic stress regulator. Normally, electrophiles can interact with proteins containing a thiol group at the cellular level, in a process called the electrophilic stress response (ESR). Glutathione, the most common molecule used by the cells against electrophilic stress, works by forming electrophile-glutathione adducts. A recent study first showed that itaconate forms adducts with glutathione [31]. The ESR is mainly sensed by the KEAP1-*Nrf2* axis *via* the reactive cysteine residues of KEAP1 and leads to the activation of *Nrf2*. Other KEAP1-independent pathways are also involved in the reaction to electrophilic stress [35]. Specifically, as every electrophilic molecule has a different extent of electrophilic properties, each has a unique ability to activate the ESR components. Accordingly, itaconate and its derivatives revealed different electrophilicity. DI showed increased electrophilicity compared with itaconate as the carboxyl group close to the double carbon-carbon bond. Supporting this, a study showed that DI could inhibit LPS-induced I*κ*B*ζ* expression. In contrast, 4-ethyl itaconate showed no effect on the level of I*κ*B*ζ* in the same model, which was attributed to the higher electrophilic properties of DI compared with 4-ethyl itaconate [31]. Apart from that, dimethyl fumarate (DMF), a strong electrophile, can exhaust GSH and activate *Nrf2* in murine BMDMs and astrocytes as well as DI, which denotes the strong electrophilicity of DI [31, 36]. Of note, DI was not metabolized to itaconate in the cytoplasm; however, DI increased the itaconate level, possibly due to its electrophilic ability or unknown receptors [37]. Therefore, the electrophilicity of DI may bring in unexpected effects that are those of the endogenous itaconate. It is necessary to emphasize the electrophilicity of derivatives when mimicking endogenous itaconate. 4-Octyl itaconate, another mimic of itaconate, is different from DI as the position of the ester group is much more distal and thus has a lower electrophilic activity than DI, which seems to make it a better candidate. Moreover, it was observed that 4-OI could be hydrolyzed to itaconate in the presence of LPS in mouse macrophages [30]. A possible mechanism is that LPS is indispensable for the expression of esterase, which can hydrolyze 4-OI to itaconate in murine macrophages [30]. Therefore, 4-OI may be more suitable for the LPS-induced inflammatory model. However, the spectrum of proteins modified by itaconate and 4-OI is still, to a certain extent, different. For instance, a study confirmed the same cysteine modification on lactate dehydrogenase A in 4-OI or LPS-treated BMDMs. In contrast, the modification of protein *γ*-IFN-inducible lysosomal thiol reductase was observed only in the 4-OI treatment group [30]. All of these data suggest that the derivatives DI and 4-OI are acceptable mimics for the study of itaconate but not perfect enough. Thus, the extra side functions of DI and 4-OI should be further explored.

## 4. Itaconate Activates the Inflammation Response and Oxidative Stress *via* the *Nrf2* Pathway

**Figure 3:**
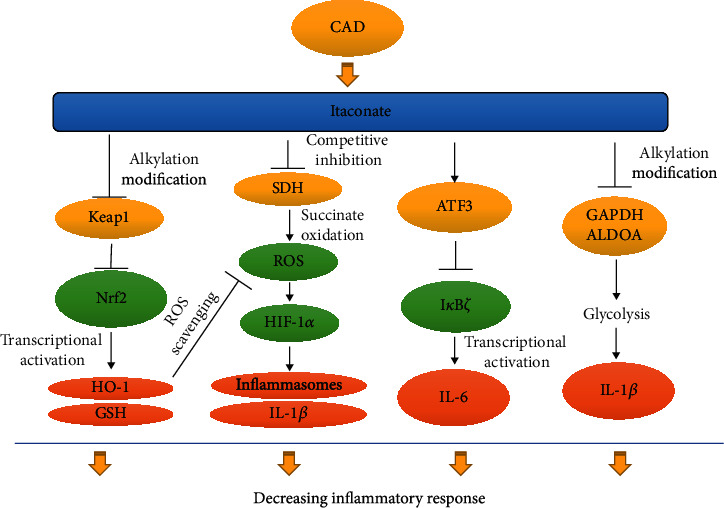
The relevant signaling pathway in itaconate-induced anti-inflammatory and antioxidative effects. Itaconate is promoted in macrophages activated by stimuli like LPS *via* increasing CAD transcription. Increasing itaconate activates the *Nrf2* pathway *via* alkylation Kelch-like ECH-associated protein 1 (Keap1), which induces the transcription of various *Nrf2*-dependent antioxidant and anti-inflammatory genes, such as *HO-1* and *GSH*. Itaconate can also inhibit succinate dehydrogenase (*SDH*) and reduce ROS generation and, consequently, IL-1*β* secretion by activation. Itaconate promotes the transcription of activating transcription factor 3 (ATF3), which directly inhibits I*κ*B*ζ* expression and leads to decreasing IL-6. In addition, itaconate directly alkylates the cysteine residue 22 of GAPDH and ALDOA to inhibit glycolysis, thereby mitigating the inflammatory response.

It is widely recognized that NRF2 is a transcriptional factor that plays a pivotal role in the regulation of the inflammatory response and oxidative stress [38–40]. The activation of NRF2 increases downstream enzymes, such as HO-1, and regulates glutathione (GSH) production, which leads to protective effects against oxidative stress (Figure 3). NRF2 induces numerous additional proteins against the cytotoxic effect, oxidative stress, and cell death. Moreover, NRF2 can directly bind to the promoter area of proinflammatory cytokine genes, such as IL-6 and Il-1*β*, and repress transcription by inhibiting the recruitment of RNA polymerase II [41]. These findings indicate that NRF2 is a multifunctional and indispensable molecule in the direct and indirect control of inflammation and oxidative stress. Under normal conditions, NRF2 combines with KEAP1 in the cytoplasm, where KEAP1 acts as an inhibitor of NRF2 [30]. Many stimuli lead to the dissociation of KEAP1 from NRF2, and NRF2 can then translocate to the nucleus to activate transcription of NRF2-dependent genes associated with anti-inflammation and antioxidation [42, 43]. Itaconate increases the alkylation of cysteine residues 151, 257, 288, 273, and 297 on KEAP1, which enhances the degradation of KEAP1 and leads to further NRF2 activation [30]. NRF2 also starts to translate antioxidant genes, such as heme oxygenase (HO-1) and *γ*-glutamyl cysteine synthase (*γ*-GCS) [30, 44]. The electrophilicity of itaconate and DI is indispensable for the activation of NRF2. A study showed that itaconate and DI directly caused electrophilic stress and reacted with glutathione, which led to NRF2 activation [31]. Recently, 4-OI was found to mitigate H_2_O_2_-induced ROS production, lipid oxidation, and cell death in SH-SY5Y cells *via* KEAP1-NRF2 signaling [45]. Another study showed that itaconate attenuated cerebral ischemia/reperfusion injury by activating the NRF2 pathway and inhibiting SDH activity [46]. Moreover, liver ischemia-reperfusion damage was mitigated by 4-OI treatment. In this study, itaconate reduced oxidative stress by activating NRF2 signaling and showed a protective effect in hepatocytes, which indicated the antioxidative properties in nonimmune cells [47]. DI also significantly reduced ROS and malondialdehyde levels in doxorubicin-induced cardiotoxicity and suppressed oxidative stress by activating the NRF2/HO-1 pathway [48]. DI treatment mitigated fungi-induced keratitis *via* NRF2 signaling by reducing inflammation [49]. 4-OI administration improved the survival rate and reduced the expression of proinflammatory cytokines in an LPS-induced sepsis mouse model [30]. The possible mechanism of this protective ability is attributed to the activation of NRF2 as *Nrf2*-/- mice displayed a poor prognosis in the sepsis model [50]. Taken together, these studies highlight the protective and antioxidant effects of itaconate that is attributed to the activation of NRF2 and consequent transcription of downstream antioxidant genes.

## 5. Itaconate Regulates Inflammation by Inhibiting SDH and the Generation of ROS

SDH, an important enzyme in the TCA cycle, converts succinate to fumarate. Itaconate was first reported in 1949 as a competitive inhibitor of SDH [51]. Initially, succinate induces the activity of hypoxia-inducible factor 1*α* (HIF-1*α*) and the production of IL-1*β* in LPS-induced macrophages [52]. The mechanism for this induction is not clarified. Then, it was realized that succinate oxidation *via* SDH could produce ROS, which enhances HIF-1*α* and, subsequently, the transcription of IL-1*β* [52]. However, the mechanism of accumulation of succinate and the inhibition of oxidation of succinate in LPS-activated macrophages was revealed only in 2016. Overexpression of *Acod1* promotes the itaconate production that leads to succinate accumulation in macrophages, as itaconate directly inhibits the activity of SDH [28, 53]. Accordingly, the administration of itaconate with BMDMs markedly increased the accumulation of succinate and decreased the consumption of oxygen [53]. Apart from explaining the relationship between succinate and HIF-1*α*, these two experiments strongly evidenced that itaconate is a specific inhibitor of SDH (Figure 3). However, itaconate is a relatively weak SDH inhibitor because a high concentration of itaconate was indispensable to inhibit SDH in an activity assay [30]. The ability of itaconate to inhibit SDH is attributed to its structural similarity to succinate. This competitive effect is similar to that of the classical SDH inhibitor malonate. Moreover, it was shown that itaconate significantly reduced LPS-induced IL-1*β* production but not TNF-*α* in murine BMDMs [28]. Also, LPS-induced ROS decreased with the administration of itaconate in murine macrophages due to the inhibition of SDH [53]. SDH can oxidize succinate and produce reduced coenzyme Q, which passes electrons to complex I and generates ROS [54]. ROS can release proinflammatory mediators by activating inflammasomes [55]. Moreover, ROS produced from complex III of the mitochondrial electron-transport chain is involved in macrophage activation and proinflammatory cytokine production [56]. And it was observed that a mitochondria-targeted ROS scavenger could also suppress LPS-induced IL-1*β* production in BMDMs [57]. Thus, inhibiting SDH is partly involved in the anti-inflammatory role of itaconate. Taken together, these studies confirmed itaconate as a metabolite that regulates the inflammatory response *via* inhibiting of SDH.

## 6. Itaconate Regulates the Activating Transcription Factor 3 (ATF3) and I*κ*B*ζ*

LPS stimulation is mediated by TLR4. The transcription of proinflammatory cytokines such as TNF-*α* and IL-6 is activated by LPS [58]. For TNF-*α* synthesis, LPS strikingly promotes the activity of the nuclear factor-*κ*B (NF-*κ*B), which directly enhances the transcription of the TNF-*α* gene [59]. However, IL-6 is induced by another pathway. I*κ*B*ζ* (also NF-*κ*B*ζ*), an ankyrin-repeat-containing nuclear protein, is encoded by the Nfkbiz gene on human chromosome 3q12.3 [60]. It was previously reported that the ablation of I*κ*B*ζ* led to a significant decrease in IL-6 production in LPS-treated mouse macrophages [61]. In addition, activation of I*κ*B*ζ* by *Streptococcus pneumoniae* markedly increased IL-6 and granulocyte-macrophage colony-stimulating factor in macrophages. Thus, I*κ*B*ζ* directly controls IL-6 production [62]. Interestingly, the administration with DI significantly decreased IL-6 secretion but not TNF-*α* in LPS-treated murine BMDMs [31]. Mechanically, DI caused conjugates of DI and glutathione due to its electrophilic properties and inhibited I*κ*B*ζ* expression [31]. This study revealed that the inhibition of IL-6 by DI might be attributed to the reduction of LPS-induced I*κ*B*ζ* activity (Figure 3). However, it was shown that the expression of I*κ*B*ζ* between *Irg1* deficient and wild-type BMDMs was similar under a single LPS administration [31]. The possible reason for this contradiction is that I*κ*B*ζ* was induced at an earlier stage than the induction of endogenous itaconate [31]. Accordingly, the second treatment of LPS could increase the I*κ*B*ζ* level in *Acod1*-deficient mouse tolerized BMDMs compared with the wild-type BMDMs [31]. Also, the discrepancy suggests that the itaconate derivatives do not fully mimic the function of the endogenous itaconate, as we discussed above.

A study revealed that the activating transcription factor 3 (ATF3) knockout increased I*κ*B*ζ* expression and enhanced the secretion of proinflammatory cytokines, which suggested that ATF3 inhibits I*κ*B*ζ* expression [63]. Further exploration found that DI was less able to decrease LPS-induced I*κ*B*ζ* expression in ATF3 negative BMDMs compared with wild-type BMDMs [31]. Moreover, the elevation of ATF3 inactivated a subunit of eukaryotic initiation factor 2 (eIF2a), which inhibited the transcription of I*κ*B*ζ* [31]. Further analysis showed that the conjugate of DI and glutathione led to a decrease of glutathione and ROS elevation. Treatment with antioxidant or cell-permeable glutathione could reduce the effect of DI-induced IL-6 expression [31]. This implied that glutathione/ROS signaling is associated with the regulation of inflammation induced by DI. As described above, Nrf2, which can also be activated by 4-OI, showed an anti-inflammatory response. However, DI can also repress IL-6 production *via* inhibiting I*κ*B*ζ* expression in an *Nrf2* knockout model [31]. Therefore, itaconate regulates the inflammatory response *via* the ATF3/I*κ*B*ζ* pathway, which is independent of the activation of Nrf2.

## 7. Itaconate Regulates the Inflammatory Response by Inhibiting Glycolysis *via* Targeting GAPDH

Glycolysis is essential for proliferation, differentiation, and function shifts in macrophages [64]. Proinflammatory stimuli such as LPS lead to the upregulation of glycolysis. The metabolic status of macrophages is associated with their immune phenotypes [64]. The energy of proinflammatory macrophages is mainly sourced from aerobic glycolysis, while the regulatory type of macrophage relies more on oxidative phosphorylation [65, 66]. The possible explanation for this is that glycolysis generates energy more swiftly, thus allowing cells to respond timely in an urgent situation like acute infections. Macrophages are artificially divided into two major types: the M1 phenotype, which is activated by LPS, has proinflammatory features, and the M2 phenotype, which is activated by interleukin-4 and interleukin-10, shows anti-inflammatory activities [67]. The metabolic hallmark of the M1 macrophage is aerobic glycolysis, which is due to a quick demand for energy to proliferate and respond rapidly to stimulation [68].

In contrast, the major energy source of M2 phenotype macrophages that proliferate infrequently is oxidative phosphorylation [69]. 4-OI inhibited the production of proinflammatory cytokines such as IL-1*β* and IL-6 in macrophages and exerted an anti-inflammatory property linked to M2 polarization [70]. However, the role of itaconate in macrophage polarization is still complex. One study reported that the decrease of *Acod1* and itaconate regulated by microRNA-93 triggered M2 polarization, possibly because the lower level of itaconate increased oxidative phosphorylation [71]. In contrast, two recent studies consecutively found that there is a negative feedback between itaconate and glycolysis, and this may be associated with the anti-inflammatory role of itaconate. One study reported that itaconate diminished the glycolysis effect by inhibiting a critical glycolytic enzyme named fructose-bisphosphate aldolase A (ALDOA) [72]. Moreover, another study found that the 4-OI treatment-induced alkylation of the Cys 22 residue of glyceraldehyde-3-phosphate dehydrogenase (GAPDH), which functionally inhibited glycolysis and the production of proinflammatory cytokines in LPS-treated murine macrophages as shown in Figure 3 [70]. The anti-inflammatory effect of 4-OI was inhibited by a high concentration of glucose, which enhances glycolysis, thus indicating that the anti-inflammatory effect of 4-OI is attributed to the inhibition of glycolysis [70]. In conclusion, these studies provided novel functional insights into the roles of itaconate in the regulation of inflammation and the relationship between itaconate and polarization of macrophages in different metabolic circumstances, but further studies are needed.

## 8. Roles of Itaconate in Cancer

Itaconate and its derivatives show protective properties in many inflammatory models. However, this anti-inflammation property may lead to adverse conditions in certain circumstances. Macrophages were found to promote the initiation, growth, and metastasis in a variety of malignancies [73]. The metabolic changes in macrophages often induce changes in the secretion of cytokines and angiogenetic factors, which lead to the progression of malignancy [74]. In a recent study, the presence of peritoneal tumors changed the metabolism of tissue-resident macrophages. The tumor cells educated tissue-resident macrophages *via* some unknown mechanisms and dramatically elevated the expression of *Acod1* and, consequently, the itaconate level as well [75]. This itaconate-rich macrophage exhibited a tumor-promoting property. Using a lentivirus to delete the *Acod1* gene and thus abolish the conversion of succinate into itaconate in tissue-resident macrophages significantly decreased the tumor volume compared with the control lentivirus group in tumor-bearing mice. Itaconate promoted tumor growth through the fatty acid oxidation-mediated increase in oxidative phosphorylation, which produced ROS [75]. ROS, which includes a wide range of molecules such as superoxide and hydroxyl radicals, affect the inflammatory response. We previously reported the ROS generation is associated with the activation of NF-*κ*B and mitogen-activated protein kinases (MAPKs), which lead to the inflammatory response [76, 77]. Moreover, ROS could activate many transcription factors, including NF-*κ*B and MAPKs, to promote the growth and survival of tumor cells [78–80]. Therefore, itaconate promoted the growth of peritoneal tumors *via* OXPHOS-induced ROS in tissue-resident macrophages, and ROS mediated the activation of MAPKs in tumor cells [75]. Also, *Acod1* expression increased CD14^+^ cells in ovarian carcinoma patients and correlated with glioma progression [81], two observations suggesting that the *Acod1*/itaconate metabolic pathway plays a pivotal role in certain types of cancers. However, several mechanisms involved in the cross talk between metabolic changes and tumor progression remain to be unraveled.

## 9. Itaconate in Sepsis

The role of itaconate in sepsis is complicated. Sepsis is defined as a life-threatening organ dysfunction caused by the dysregulation of the host response to infections [82]. The mechanism underlying sepsis is not fully clarified. The unbalance of the increasingly inflammatory response and immunosuppression is one of the crucial factors in the development of sepsis. Historically, the poor prognosis was attributed to an excessive inflammatory response. However, therapeutic interventions aimed at inhibiting inflammatory responses such as glucocorticoids, and anti-TNF-*α* antibodies failed to improve the outcomes in septic patients [83]. Currently, a single administration of anti-inflammatory agents is considered not valuable in the treatment of sepsis. 4-OI protected mice from LPS-induced death and inhibited the inflammatory response [30]. LPS is widely used to set up sepsis in a murine model [84]. Moreover, HO-1, which generates endogenous carbon monoxide (CO), induced *Irg1* expression and production of itaconate, leading to the inhibition of the inflammatory response [85]. Therefore, itaconate exerts a certain protective effect in the sepsis model by regulating the excessive inflammatory response [70]. However, in sepsis, itaconate may play an adverse role, termed immunoparalysis [86]. Immunoparalysis is the lack of an immune response during sepsis, from which it is very difficult to recover and which could lead to susceptibility to the second infections and increasing mortality [86]. Macrophages that were initially stimulated with LPS showed a strong response to LPS. Those macrophages displayed a significantly weaker response following the second treatment of LPS and were termed “tolerized macrophages” [31]. This type of macrophage may be beneficial to relieve tissue damage. However, an insufficient response to an inflammatory stimulus leads to susceptibility to secondary infections, which is lethal in sepsis [86]. Itaconate is possibly involved in this innate immune tolerance as the mRNA level of *Irg1* is much higher in monocytes obtained from postacute septic patients compared with healthy donors [87]. In contrast to immunoparalysis, trained immunity is a process that can resist immunological paralysis by upregulating immune responses in innate immune cells to the secondary pathogen stimulation following the initial exposure to a stimulus. Of note, the *β*-glucan can mobilize tolerized macrophages to react with secondary LPS stimulation by inhibiting *Acod1* expression and elevating the SDH activity. Pretreatment DI can inhibit secretion of TNF-*α* and IL-6 in *β*-glucan-activated monocytes following a secondary stimulus of LPS, which indicates that the itaconate pathway is involved in immunoparalysis in sepsis [88]. Therefore, the production of itaconate may play different roles in different stages in sepsis, and its functions still need further studies.

## 10. Future Therapeutic Role of Itaconate

Itaconate showed marked anti-inflammatory and antioxidative properties in many animal models *via* the regulation of many signaling pathways, including Nrf2 and ATF3, which indicated a feature similar to other Nrf2 activators. The Nrf2 pathway is pivotal in the regulation of oxidative stress and inflammation [46]. Certain agonists of Nrf2 have already been established to be effective in the treatment of some inflammatory diseases. For example, dimethyl fumarate was validated to be efficient in the activation of Nrf2 and applied in the clinical treatment of multiple sclerosis [89]. Also, a clinical study showed a lower relapse rate after remission than a placebo in multiple sclerosis patients [89]. In addition, itaconate is an endogenous metabolite, and thus, toxicity should be quite low for therapeutic use. Therefore, itaconate has a high potential for clinical use in treating inflammatory-associated diseases with low side effect potency. However, further studies are needed as there are several lingering concerns about itaconate. Currently, there is still no clear evidence on the consequences of the *in vivo* deletion of itaconate and no reports showing if there is an autoimmune response when itaconate is lacking. The long-term detrimental effects of the absence of itaconate should be explored before any tentative therapeutic strategic is used.

The other potential therapeutic effect of itaconate lies in the link of its metabolism and vitamin B12 [25]. The lack of citramalyl-CoA lyase (CLYBL), which converts citramalyl-CoA to acetyl-CoA, leads to the diminishing of vitamin B12 in the serum and accumulation of itaconyl-CoA [25]. The mechanism underlying the decrease in vitamin B12 is still not fully understood. Until recently, itaconyl-CoA was demonstrated that it could form adduct with 5′-deoxyadenosyl moiety of the B12 coenzyme and inhibited activity of methylmalonyl-CoA mutase [90]. All these provided a link between itaconate a4nd vitamin B12. LPS treatment significantly reduced vitamin B12 levels and increased itaconyl-CoA levels (intermediate of itaconate metabolism) in macrophages. Immune activation-induced itaconate production leads to vitamin B12 deficiency [25]. Thus, the anti-inflammatory property of itaconate may be mediated by vitamin B12. Given that CLYBL deficiency is common in humans (2.7% lost) and well-tolerated, targeting CLYBL is a promising strategy to simulate itaconate downstream effects [25]. This study reveals the complexity of itaconate functions and discloses an alternative angle for clinical use. Moreover, the quantitative trait locus mapping of single nucleotide polymorphism in *Acod1*/*Irg1* could be promising in the future studies, which may provide connections and understandings between *Acod1* gene and certain diseases.

## 11. Conclusion

Itaconate was found in the 18th century, but unfortunately, only recently its immunoregulatory functions have been explored. Since its pivotal immunoregulatory roles were revealed in macrophages, we began to realize the complex interaction between the metabolism and immune response, and this gave us a novel perspective on inflammatory diseases. Currently, we know that itaconate can reprogram murine macrophages into an M2-like phenotype with a significant inflammation-resolving ability. The anti-inflammatory and antioxidative mechanisms include the activation of Nrf2 by releasing from Keap1, inhibition of SDH, induction of ATF3 to repress the activation of I*κ*B*ζ*, and downregulation of glycolysis *via* alkylation of GAPDH and inhibition of ALDOA. Moreover, the electrophilicity of itaconate and its derivatives is indispensable in the immunoregulating process. All of these novel findings highlight itaconate as a very promising therapeutic target to limit the pathological consequences of inflammatory diseases. However, currently, most outcomes of itaconate are from animal models or *in vitro* studies. The underlying immunoregulatory mechanisms need to be fully clarified before starting clinical experiments. Furthermore, side effects, such as the promotion of specific tumor types and induction of immune paralysis, still need more studies. In summary, itaconate is a very promising therapeutic target for treating inflammatory diseases, but additional analyses are required for the development of a novel immunoregulatory drug.

## Abbreviations

TNF: Tumor necrosis factors
IL: Interleukin
ROS: Reactive oxygen species
TCA: Tricarboxylic acid
LPS: Lipopolysaccharide
*Acod1*: Aconitate decarboxylase 1
TLR: Toll-like receptor
SDH: Succinate dehydrogenase
Nrf2: Nuclear factor E2-related factor 2
*γ*-GCS: *γ*-Glutamyl cysteine synthase
BMDMs: Murine bone marrow-derived macrophages
DI: Dimethyl itaconate
4-OI: 4-Octyl itaconate
Keap1: Kelch-like ECH-associated protein 1
HO-1: Heme oxygenase
GSH: Glutathione
ATF3: Activating transcription factor 3
GAPDH: Glyceraldehyde-3-phosphate dehydrogenase
CLYBL: Citramalyl-CoA lyase
HIF-1*α*: Hypoxia-inducible factor 1*α*
*Irg1*: Immune response gene 1
IGF: Insulin-like growth factor
IFN: Interferon
CD: Cluster of differentiation
ATP: Adenosine triphosphate
CAD: Cis-aconitate decarboxylase
ESR: Electrophilic stress response
DMF: Dimethyl fumarate
NF-*κ*B: Nuclear factor-*κ*B
MAPKs: Mitogen-activated protein kinases
OXPHOS: Oxidative phosphorylation
ALDOA: Fructose-bisphosphate aldolase a
I*κ*B: Inhibitor of NF-*κ*B
CO: Carbon monoxide.
